# Nanostars planarity modulates the rheology of DNA hydrogels†

**DOI:** 10.1039/d2sm00221c

**Published:** 2023-05-25

**Authors:** Yair Augusto Gutiérrez Fosado

**Affiliations:** a School of Physics and Astronomy, University of Edinburgh, Peter Guthrie Tait Road Edinburgh EH9 3FD UK yair.fosado@ed.ac.uk

## Abstract

In analogy with classic rigidity problems of networks and frames, the elastic properties of hydrogels made of DNA nanostars (DNAns) are expected to strongly depend on the precise geometry of their building blocks. However, it is currently not possible to determine DNAns shape experimentally. Computational coarse-grained models that can retain the correct geometry of DNA nanostars and account for the bulk properties observed in recent experiments could provide missing insights. In this study, we perform metadynamics simulations to obtain the preferred configuration of three-armed DNA nanostars simulated with the oxDNA model. Based on these results we introduce a coarse-grained computational model of nanostars that can self assemble into complex three dimensional percolating networks. We compare two systems with different designs, in which either planar or non-planar nanostars are used. Structural and network analysis reveal completely different features for the two cases, leading to two contrasting rheological properties. The mobility of molecules is larger in the non-planar case, which is consistent with a lower viscosity measured from Green–Kubo simulations in equilibrium. To the best of our knowledge, this is the first work connecting the geometry of DNAns with the bulk rheological properties of DNA hydrogels and may inform the design of future DNA based materials.

## Abstract 2

In analogy with classic rigidity problems of networks and frames, the elastic properties of hydrogels made of DNA nanostars depend on the precise geometry of their building blocks and the topology of the network.

## Graphical abstract

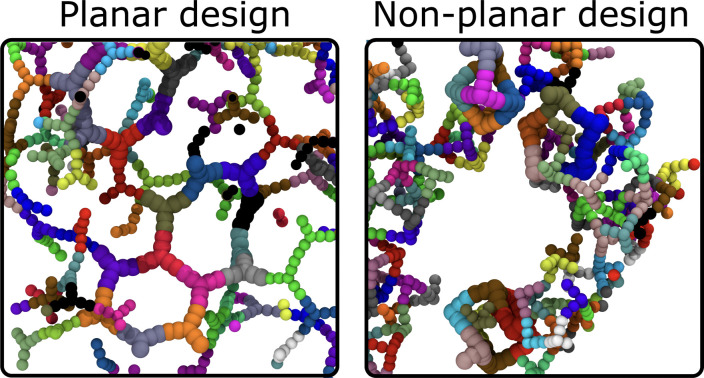

## 1. Introduction

The specific binding rules through which DNA nucleotides form pairs (Adenine–Thymine and Guanine–Cytosine) has been long known to be key for the storage and replication of the genetic information. This same mechanism is employed in DNA nanotechnology to form DNA motifs, *i.e.*, artificial structures with high programmability. Here we study DNA nanostars (ns), motifs made by several double-stranded (ds) arms connected into a single structure.^1,2^ The number of arms per DNAns defines its valence (*f*). Each of these arms is provided with a sticky end which, under appropriate conditions, allows nanostars to hybridize into complex three dimensional percolating networks to form a DNA hydrogel.^3^ It is due to their inherent biocompatibility, besides the possibility of functionalization^4^ and systematic control over their mechanical properties,^5–7^ that hydrogels have emerged as promising materials in the development of diverse applications such as biosensing,^8^ drug delivery^9^ and tissue engineering,^10^ among others.

In recent years, there have been very successful studies to characterize the properties of DNA hydrogels. In reference,^11^ for example, it was suggested that key components for the formation of these materials were the limited valence^12,13^ and the internal flexibility of DNAns.^14–17^ The phase diagram of gels made of DNAns with *f* = 3 is reported in ref. 3. In ref. 18, microrheology experiments of trivalent DNAns were performed at very large concentrations (20 mg mL^−1^), at which the system exhibits a phase transition from a fluid of disconnected DNAns, at high temperatures, to a fully bonded state with maximum network stiffness at low temperature. It was also proved that a flexible section in the vicinity of the sticky ends (as conferred by unpaired bases in the DNAns design) produces gels with lower bulk elasticity. The role of valence was tested in ref. 19 by oscillatory bulk rheology of gels made from DNAns designs with different number of dsDNA arms (ranging from three to six). It was found that at the same concentration of DNAns, the higher the nanostar valence the stiffer the network formed. Finally, viscoelastic properties of tetravalent DNAns at different salt concentration were investigated in reference.^20^

The previous results expose an important aspect of DNA hydrogels: that beyond base-pairing thermodynamics, the precise topology of DNAns is a key component that determines the elastic properties of networks.^21–23^ Understanding the relationship between the geometry of nanostars and the macroscopic mechanical properties of DNA hydrogels is essential and a subject of ongoing study. Computer simulations can shed light into this aspect. Indeed, simulations with the oxDNA^24^ model of a system of tetravalent DNAns have been performed in the past.^25–28^ These simulations yield to a phase diagram in fair agreement with experimental results^3^ and provided the first strong evidence that gels of DNAns should not crystallize. However, the level of coarse-graining and specificity in the oxDNA model, would make computationally unfeasible to simulate a system comprising more than 100–200 nanostars, not to mention, exploring the role of different geometrical designs. Models adopting a lower resolution are therefore needed, yet they need to be inferred from higher resolution ones.

Recently, a bead-spring coarse-grained model of trivalent DNAns was introduced in ref. 29. In this model, each nanostar is represented by ten beads arranged into a Y-shaped planar structure. Adjacent nanostars can hybridized through specific binding sites, capturing in this way the overall network formation. The model represents an important step in the study of thermodynamic, structural and rheological features of percolating networks. However, since experiments cannot resolve the detailed geometry of nanostars, their shape is usually assumed to be planar in this type of simulations.

Here, we propose a new way of inferring the geometry of a single DNAns from metadynamics simulations of higher resolution models. This biasing technique allows the quick inspection of DNAns conformations by flattening the free energy landscape. From this computation we characterize how the planarity of DNAns is affected by introducing modifications in their design. Based on this, we then build a coarse-grained model of rigid analogues of DNAns and we compare the structural features, melting response and linear elasticity of two networks made of either, planar or non-planar molecules.

## 2. Nanostars design

**Fig. 1:**
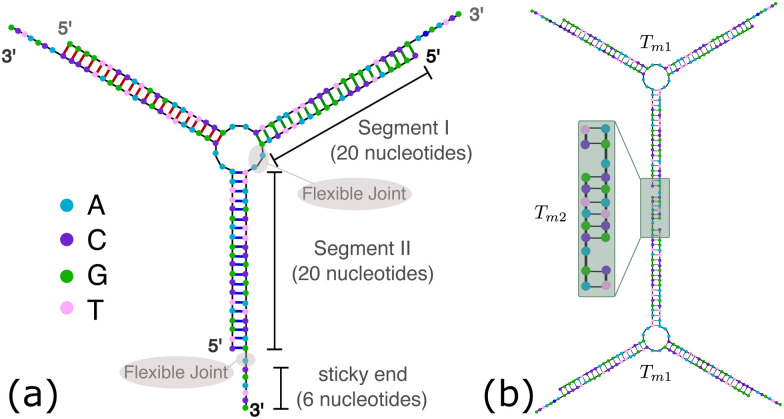
DNA nanostars design. (a) Schematic representation of the ssDNA oligonucleotides assemble into a DNAns. Different nucleotides are identified through a colour code: adenine (A) cyan, cytosine (C) purple, guanine (G) green and thymine (T) in pink. The directionality of the chains, from 5′ to 3′, is indicated in the first and last nucleotides of each ssDNA. Labels of the five functional parts described in Table 1 (two flexible joints, the sticky end and the two segments) are shown for one of the strands. Backbones are depicted in black and hydrogen bonds are coloured according to Table 1. (b) Cartoon of the hybridization between two DNAns connected through the sticky ends. Sequences used here ensure that the melting temperature of the network (*T*_m2_) is lower than the melting of individual stars (*T*_m1_).

OxDNA is a well established single nucleotide resolution model that is based on force fields tuned to account for several geometrical and thermodynamic features of single and double stranded DNA. Here we use its most recent implementation^30^ into the LAMMPS^31^ engine, in order to simulate DNA nanostars made of three single-stranded (ss) oligonucleotides. Sequences are reported in Table 1 and are similar to the ones used in ref. 19. Each ssDNA is 49 bases long and consists of five regions. The segments I and II (20 nucleotides long each) are designed to form the three dsDNA arms. In between the two segments there are two A-nucleotides acting as a spacer and forming the flexible joint at the nanostar core, (FJC). The sticky end is formed by 6 self-complementary bases and has the same sequence for the three oligonucleotides, allowing in this way the non-specific hybridization of two nanostars: any of the three arms of one ns can hybridize with any (but only one) of the arms of another ns. Finally, there is a second flexible joint (FJ), formed by an A-nucleotide in between segment II and the sticky end. Fig. 1(a) and (b) show schematic representations of the DNAns assemble and the binding of two nanostars, respectively. Beyond structural features, the overall sequences employed provide certain stability. The melting temperature (*T*_m1_) of individual stars is larger than the melting temperature (*T*_m2_) of the hybridization between stars. This ensures that there is a range of temperatures (below *T*_m1_) in which the core structure of nanostars does not suffer major changes and the assemble/break-up of the network can be proved. This is the regime considered in the present work.

**Table 1:** Strand sequence used in the nano star design with valence *f* = 3 and *n* = 2 unpaired nucleotides at FJC. Each row represents a different ssDNA oligonucleotide. Segments with the same colour have complementary sequences to form the double stranded arms as shown in Fig. 1

Segment I	FJC	Segment II	FJ	Sticky end
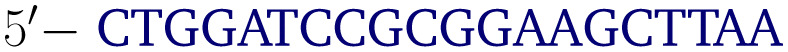	AA	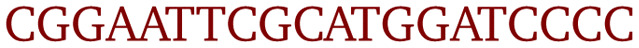	A	CGATCG-3′
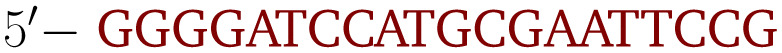	AA	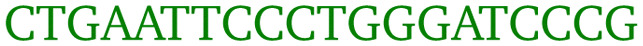	A	CGATCG-3′
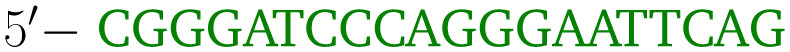	AA	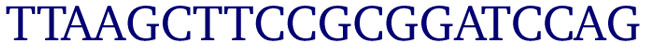	A	CGATCG-3′

## 3. Planarity of DNAns from metadynamics simulations

Molecular dynamics (MD) simulations are often used to assist the design of DNA nanostructures. However, in scenarios where the free energy landscape (FEL) of the system is complex, with several local minima separated by large energetic barriers, it becomes difficult to ensure that in the course of the simulation the phase-space to move from one minimum to the next one has been completely explored. Metadynamics^32,33^ is a computational method that provides a framework to determine free energies and accelerate rare events, allowing the system to escape from local minima in the FEL. In essence, in the metadynamics simulations we need to find a set of collective variables (CVs), *ζ*_*i*_(**r**), that gives relevant information about the state of the system and that it only depends on the position, **r**, of particles. Then, the system is biased to explore different regions on the phase-space by adding a history-dependent Gaussian potential *U*_G_(*ζ*_*i*_(**r**),*t*). The basic assumption of metadynamics is that after a sufficiently long time, *U*_G_ provides an estimate of the free energy landscape *F*(*ζ*_*i*_). A more detailed explanation of this technique is provided in the ESI.†

**Fig. 2:**
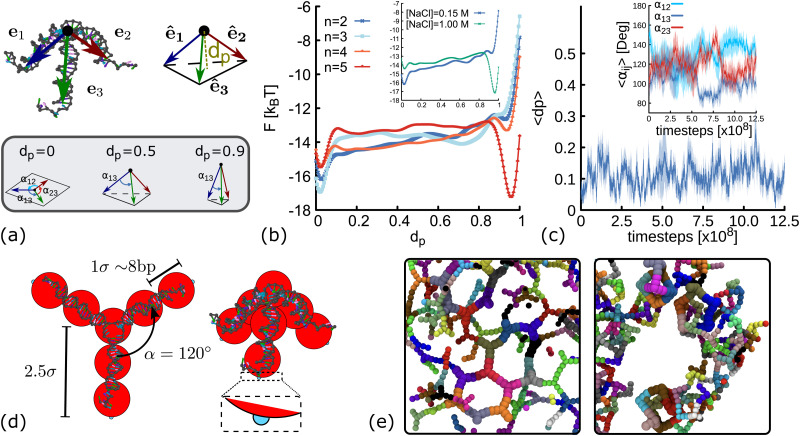
Coarse-grain modelling of DNA nanostars. (a) Schematic representation of *d*_p_ and *α*_*ij*_. Top panel shows the vectors **e**_1_, **e**_2_, **e**_3_ pointing from the core of the molecule (black dot) to the last base-pair in each arm. The variable *d*_p_ represents the distance from the core of the molecule to the plane touching the end of the normalized vectors **ê**_1_, **ê**_2_, **ê**_3_ (see ESI† for details). Bottom panel shows sketches of three DNAns with different degree of planarity, from left to right the value of *d*_p_ is 0, 0.6 and 0.9. (b) Free energy landscape as a function of *d*_p_ for DNAns simulated with the oxDNA model at [NaCl] = 0.15 M. Different colours represent results for different number of unpaired nucleotides at the FJC. Inset shows comparison for *n* = 2 at two salt concentrations. (c) Time evolution of *d*_p_ and *α*_12_, *α*_13_, *α*_23_ (averaged over five different replicas) obtained from MD simulations of a DNAns with the oxDNA model. Shaded area enveloping the curves is the error of the mean. (d) Coarse-grained geometries of planar (left, with *d*_p_ = 0) and non-planar (right, with *d*_p_ = 0.6) molecules. Red beads represent the dsDNA sections of the molecules. Small patches at the end of each arm are displayed in cyan. (e) Snapshots from simulations of networks formed when using planar (left) and non-planar (right) DNAns.

The method described above is used here to obtain the FEL as function of *d*_p_, a collective variable related to the degree of planarity of a DNAns. This variable is defined as the magnitude of the vector pointing from the core of the molecule to the plane touching the tip of the three unitary vectors representing the direction of the three dsDNA arms (see Fig. 2(a) and ESI†). The lower the value of *d*_p_ the more planar the molecule. Fig. 2(b) shows the FEL of DNAns designs with a varying number (*n*) of unpaired nucleotides at the core of the molecule, when the salt concentration of the system is [NaCl] = 0.15 M. For *n* = 2 there is a global minimum located at *d*_p_ ∼ 0 (planar), with an energy barrier to overcome before exploring other regions. This indicates that the planar configuration of the nanostars would be the most favourable. As *n* increases (for instance when *n* = 3), the energy barrier becomes lower and two minima are displayed. This suggests the coexistence of two different conformations with *d*_p_ ∼ 0 and *d*_p_ ∼ 0.7, indicating that in a solution of DNAns, a fraction will be in a non-planar metastable configuration. For *n* = 5 the free energy exhibits two clear minima, with the global one favouring the non-planar configuration of the nanostar. The trajectory provided in the Movie S1 (ESI†) shows that in this case, intramolecular bonds are formed, explaining the deep minimum at *d*_p_ ∼ 1. This is an interesting feature that would have been difficult to observe using normal MD simulations. We interpret the results from metadynamics in the following way: for small values of *n*, each dsDNA arm is constrained by the presence of the other two and non-planar configurations will create stress, particularly at the core of the molecule. As *n* increases, the dsDNA arms become more disconnected and more configurations are accessible.

In the inset of Fig. 2(b) we compare the FEL of DNAns with *n* = 2 at two different salt concentrations. The position of the global minimum changes from *d*_p_ ∼ 0 (planar) to *d*_p_ ∼ 1 (highly non-planar) as [NaCl] changes from 0.15 M to 1 M. This behaviour occurs because at high salt concentrations the electrostatic repulsion within the DNAns is screened. Therefore, the effective diameter of the dsDNA arms decreases,^34^ facilitating the non-planar configuration of the DNAns and the formation of intra-molecular bonds.

To infer the angle between arms in a single DNAns, we performed long molecular dynamics simulations (without any bias) in conditions favouring the planar design of the molecule: [NaCl] = 0.15 M, *T* = 300 K and *n* = 2. We let the system equilibrate for 2.5 × 10^8^ timesteps, and then perform a production run where we measure *d*_p_ and the angles *α*_*ij*_ = cos^−1^**ê**_*i*_·**ê**_*j*_ between arms *i* and *j* of five different replicas (see Fig. 2(a)). Results are reported in Fig. 2(c) and suggest that nanostars spend most of the time in a planar configuration (*d*_p_ ∼ 0.1 ± 0.1), resembling a Y-shaped structure, with angles between arms fluctuating around *α*_*ij*_ ∼ 120° ± 20°. An example of one of the trajectories is provided in the Movie S2 (ESI†).

The previous results suggest that minor changes in the design of the DNAns have major implications on their shape. In particular, unpaired bases at the core and changes in salt concentration affect their planarity. Furthermore, our FEL indicates that for some designs, the planarity of the DNAns fluctuates over time, yielding to a state in which both planar and non-planar molecules coexist in solution. To investigate what is the effect of DNAns geometry in the bulk gel properties, here we introduce a coarse-grained model of rigid analogues of trivalent nanostars (Fig. 2(d)) and we study a monodisperse system made of only planar or only non-planar molecules. In this model, each nanostar is represented by a rigid body made of ten particles. Seven beads (depicted in red in Fig. 2(d)) represent the core of the molecule and the three dsDNA arms. Attractive patches (depicted in cyan) are placed at the edge of the last bead in each arm, mimicking in this way the sticky ends interactions. Beads have an excluded volume of *σ* = 2.5 nm ∼ 8 bp, so nanostars cannot overlap. Patches interaction is set *via* a Morse potential with energy *ε*_m_ that ensures the attraction of patches in a radius of 0.2*σ* (see ESI† for details). In the planar case (*d*_p_ = 0), the angles between arms of the same DNAns are set to *α*_*ij*_ = *α* = 120°, the preferred value found in simulations. In the ESI† we also corroborate that the general results hold when fluctuations of *α*_*ij*_ are allowed in the model. In the non-planar case we use an intermediate value of *d*_p_ = 0.6, the geometry in the coarse-grain model is the one of a tetrahedron with an equilateral triangle base and three equal isosceles triangle sides. We also show in Section 6 below that results are consistent across different values of *d*_p_ ∈ [0.1,0.9]. It should be noted that these non-planar configurations force the angles between arms *α*_*ij*_ < 120°. For instance, in the extreme and unrealistic case in which the three dsDNA arms are colinear (*d*_p_ = 1), *α*_*ij*_ = 0°. We also note that with the rigid model described here, the self hybridization of the arms of a DNAns that results into an intra-molecular bond cannot be proved.

Fig. 2(e) shows snapshots from simulations of networks formed when using planar and non-planar molecules. Clear differences in the shape and connectivity of the networks can be seen. In the following sections we investigate more in detail these differences and how they affect the elastic properties of the networks.

## 4. Melting curves and relaxation time

We first study the formation of the network *via* molecular dynamic simulations employing the model previously described (see ESI† for more details on the model and the MD). In the simulations reported here, we start from an equilibrated configuration of *N* = 175 unconnected nanostars, with *ε*_m_ = 0 and temperature *T*. The system is in a cubic box of size *L* = 40*σ* such as the volume fraction is *ρ* = 0.01 (in Section S7 and ESI† we also show results at *ρ* = 0.06, with *N* = 1050 nanostars). Then, we turn on the morse attraction between patches, and record the time evolution of the system until a steady state is reached. It is worth mentioning here that in the coarse-grained model we quote time in units of the Brownian time (*τ*_Br_), which is proportional to the time needed for a bead to diffuse its own size: *τ*_Br_ = *σ*^2^/*D* = 3π*η*_s_*σ*^3^/*k*_B_*T*, with *D* the diffusion coefficient of the bead and *η*_s_ the viscosity of the solvent.

**Fig. 3:**
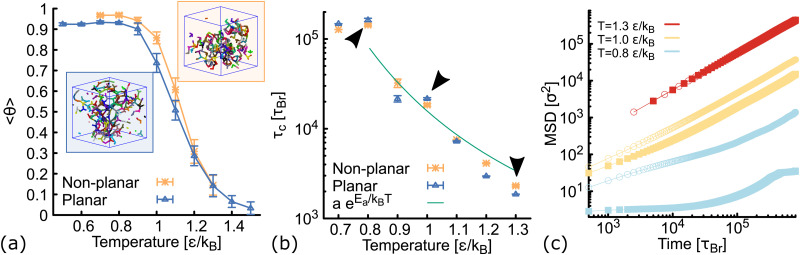
Results from simulations at different temperatures of systems comprising either planar (blue) or non-planar (orange) molecules. (a) Melting curves obtained *via* MD simulations from the averaged number of connected patches at long times, (see Fig. S3 and ESI†). Note that the melting temperature (*T* ∼ 1.15*ε*/*k*_B_) of non-planar molecules is larger than the one (*T* = 1.1*ε*/*k*_B_) for planar molecules. Insets show snapshots of typical configurations obtained in simulations. (b) Log-linear plot of the relaxation time of the networks, obtained from the autocorrelation function of *N*_c_(*t*) (see Fig. S3, ESI†). The green line represents the fit to the data using an exponential function with the general form *a*e^*E*_a_/*k*_B_*T*^. From the fit we obtain *a* = 0.8*τ*_Br_ and *E*_a_ = 9.7 *k*_B_*T*, which is compatible with the depth of the Morse attractive potential set in simulations (see ESI†). Black arrows indicate the temperatures related to the MSD shown in the next panel. (c) Log–log plot of the MSD of DNAns for planar (filled squares) and non planar (open circles). Colours represent results at different temperatures: below (*T* = 0.8 *ε*/*k*_B_ in blue), close (*T* = 1.0 *ε*/*k*_B_ in yellow) and above (*T* = 1.3 *ε*/*k*_B_ in red) the melting temperature.

**Fig. 4:**
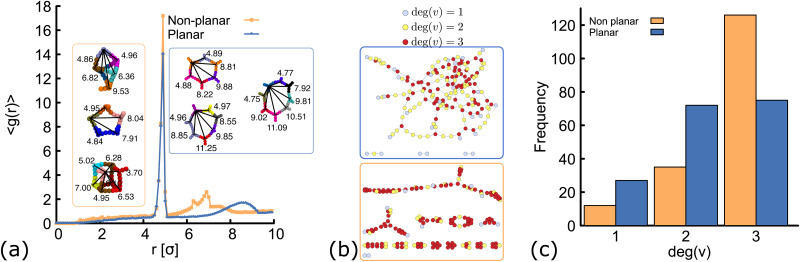
Network structure of planar (blue) and non-planar (orange) molecules. All the data shown here correspond to simulations performed at *ρ* = 0.01 (details and results at different temperatures and concentrations can be found in the ESI†, Fig. S3 and S6). (a) Radial distribution function computed from simulations at *T* = 1.0*ε*/*k*_B_. In the insets, typical geometries found in simulations of the two networks are shown. In each “ring” structure, black lines connect the core of two DNAns and the labels show the distance (in simulation units) between them. The top-most ring shown inside the orange and blue rectangles, correspond to rings made of six DNAns in the non-planar and planar cases. While in the former there are eight contacts between neighbouring DNAns, in the latter there are only six contacts. (b and c) Network diagrams and histograms showing the connectivity between nanostars.

A melting curve reports the dissociation of the dsDNA into ssDNA after a change in the solution conditions (*e.g.* increase in temperature), from the absorbance of ultra-violet light passing through a solution of DNA. An observable that can be directly compared with these experiments is the fraction of connected DNAns, *θ* = 2*N*_c_(*t*)/*Nf*, with *N*_c_(*t*) the total number of contacts between patches at time *t*. The plot of the equilibrium value 〈*θ*〉 as a function of temperature is identified with the melting curve of the system. This is reported in Fig. 3(a) for networks formed with planar and non-planar molecules. At high temperatures 〈*θ*〉 → 0, indicating that patches are dissociated and resembling a gaseous state for the two systems. As temperature decreases, DNAns bonds start to form, but the melting temperature (at which 〈*θ*〉 = 0.5) is larger for the network made of non-planar molecules. At low temperatures 〈*θ*〉 plateaus at a value close to 1 for both systems. The networks have formed all the possible bonds, but the non-planar molecules present a consistent higher fraction of connections. As we will show in the next section, this effect is related to the geometry of DNAns (see Fig. 4(a)).

The characteristic time (*τ*_c_) for network reconfiguration,^35^*i.e.*, the time that it takes for one of the DNAns to unbind and bind somewhere else, can be measured from the autocorrelation function of *N*_c_(*t*) (see ESI†). Values obtained at different temperatures are reported in Fig. 3(b). As expected, at high temperatures thermal fluctuations facilitate the unbinding of patches and in consequence *τ*_c_ is small. At low temperatures instead, thermal fluctuations are weaker and therefore, the relaxation time of the network increases. We note that *τ*_c_ plateaus at *T* ≤ 0.8 *ε*/*k*_B_, indicating that fluctuations of *N*_c_(*t*) in this range of temperatures are very similar.

It has been shown^19^ that *τ*_c_ exhibits an Arrhenius dependence: *τ*_c_ ∝ e^*E*_a_/*k*_B_*T*^, where *k*_B_ is the Boltzmann constant and *E*_a_ is associated to the binding energy of the sticky end. A fit to our data using this equation is depicted by the green line in Fig. 3(b). The agreement is reasonable, considering the simplicity of our model. Remarkably, at low temperature *τ*_c_ is larger for the planar case and, as the temperature increases, this difference becomes smaller. This is in agreement with the results of the mean squared displacement of the centre of mass of the nanostars (MSD(*t*) = 〈[*r*_CM_(*t* + *t*_0_) − *r*_CM_(*t*_0_)]^2^〉, where the average is performed over nanostars and *t*_0_), reported in Fig. 3(c). At *T* = 0.8 *ε*/*k*_B_, the network made of non-planar molecules shows a larger mobility. The difference in mobility decreases at *T* = 1.0 *ε*/*k*_B_ and it becomes negligible at *T* = 1.3 *ε*/*k*_B_, when both systems are fully disconnected.

## 5. Structural analysis

In order to understand the structure of the networks formed in our simulations, we first analyse the results obtained at constant temperature (*T* = 1.0*ε*/*k*_B_) and volume fraction (*ρ* = 0.01). We compute the radial distribution function (RDF), *g*(*r*), using the position of the beads at the core of the molecules and averaging over configurations in the steady state (see ESI† for details). Results are depicted in Fig. 4(a). Both networks, made of planar and non-planar molecules show a global maximum of *g*(*r*) located close to *r* = 5 *σ*. This corresponds to the average distance between the cores of two bound nanostars. A peak is located in between 7.5 < *r* < 9 *σ* in the planar case, with local maximum at *r* = 8.5 *σ* (distance between second nearest neighbours). By contrast, in the non-planar case there are two distinct local maxima located at *r* = 6.4 and 6.9*σ*, corresponding to the distance between second and third nearest neighbours, respectively. These results are consistent with the ring structures observed in simulations and depicted in the insets of Fig. 4(a). Remarkably, simulations displayed a rich variety of unanticipated structures. For example, while rings made of only six DNAns are expected in the planar case (because *α* = 120°), we found some rings made of seven, eight or even more DNAns. In Section 6 we show that there is an effective bending angle between the arms of two connected DNAns (so they are not completely aligned), which is related to the configurations found. In the case of non-planar molecules, not only rings are formed but also box-like structures. It is worth noting here that if we compare, for example, rings made of six DNAns in the insets of Fig. 4(a), eight contacts are made between neighbouring DNAns in the non-planar case and only six contacts in the planar case. This result explains why 〈*θ*〉 is consistently larger in Fig. 3(a) and suggests that the non-planarity of the molecules would affect the degree of connectivity of DNAns in the network as we will see below.

In Fig. 4(b) we show diagrams of the connection between DNAns in the network. In this network diagrams, each DNA nanostar is represented by a circle (also called vertex). A line (also called edge) is drawn between any two connected nanostars and colours are used to represent the degree of a vertex (deg(*v*)), *i.e.*, the number of DNA nanostars connected to that vertex. Because the DNAns valence is *f* = 3, the value of deg(*v*) can be either: 0 (for isolated stars, not shown in the plot), 1 (light-blue circles), 2 (yellow circles) or 3 (red circles). The histogram depicting the frequency of the nanostars with certain degree of connection is also shown in Fig. 4(c). As it can be seen, the number of DNAns fully connected (deg(*v*) = 3) is larger for the non-planar molecules. This is also reflected in the higher density of red circles in the network diagram at the bottom, which would explain why 〈*θ*〉 in Fig. 3(a) is smaller for the planar network.

In the network diagrams, a connected component is a set of vertices with edges spanning paths to connect any two of them. The larger the set of vertices in a component, the higher the degree of connectivity in the system. By inspecting the network diagrams in Fig. 4(b), it is evident that in the planar system most of the DNAns participate in the network and a few of them form small clusters. On the other hand, the non-planar system shows several clusters. Therefore, the degree of connectivity is larger for the planar case. One way to show this, is by computing the number (*c*_s_) of DNAns that are part of the largest component of the network (normalized by the total number of DNAns, *N*). This is reported in the ESI† (Fig. S4) for different temperatures, and shows that results are consistent across the range of temperatures explored here. The implications of these observations on the rheology of the networks are explored in Section 7, but before let's study the effect of the degree of planarity in the connectivity of the network.

## 6 Varying *d*_p_

**Fig. 5:**
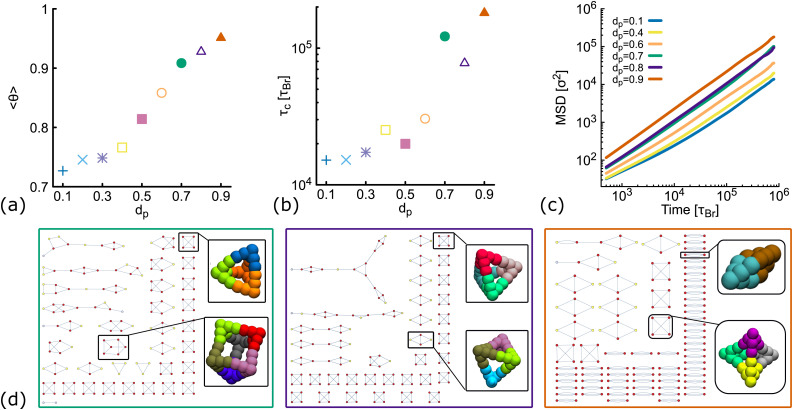
Results from simulations of DNAns with different degree of planarity at *ρ* = 0.01 and fixed temperature *T* = 1.0*ε*/*k*_B_ (see also Fig. S5 in the ESI†). Different colours represent different planarity: from *d*_p_ = 0.1 to *d*_p_ = 0.9. In orange we show results for *d*_p_ = 0.6, the one used so far as non-planar in the main text. (a and b) Show the fraction of contacts *(θ*) and the relaxation time *(τ*_c_) as function of *d*_p_. (c) Shows log–log plots of the MSD averaged over all the molecules in the system. (d) Network diagrams showing the connectivity between DNAns at the last timestep from simulations. From left to right we display results for *d*_p_ = 0.7, 0.8, 0.9. In each case, examples of box-like structures formed by the DNAns are shown.

So far, we have considered non-planar nanostars with a fixed value of *d*_p_ = 0.6. Here, we check that results are consistent for networks formed by DNAns with a different degree of planarity. Simulations are performed at a fixed temperature (*T* = 1.0*ε*/*k*_B_) and concentration (*ρ* = 0.01). Fig. 5(a) shows the equilibrium value of the fraction of contacts as function of *d*_p_. We observe that as *d*_p_ increases, more contacts between DNAns are formed, which is consistent with an increase in the relaxation time of the network (Fig. 5(b)). In the MSD reported in Fig. 5(c) we observe that the larger the value of *d*_p_ the higher the mobility of the system. These results are all in agreement with the details presented in the previous sections.

The network diagrams for simulations performed at *d*_p_ = 0.7,0.8 and 0.9 are depicted in Fig. 5(d). We note that for less planar designs (higher *d*_p_), although DNAns form more contacts, they are also less connected and more clusters made by four or fewer DNAns appear in the diagrams. Importantly, the design with *d*_p_ = 0.9 is the only one for which a DNAns can form two or three bonds with the same neighbouring nanostar. In fact, the right-most diagram shows a preference of the system to form dimers and in this way to increase the number of contacts.

**Fig. 6:**
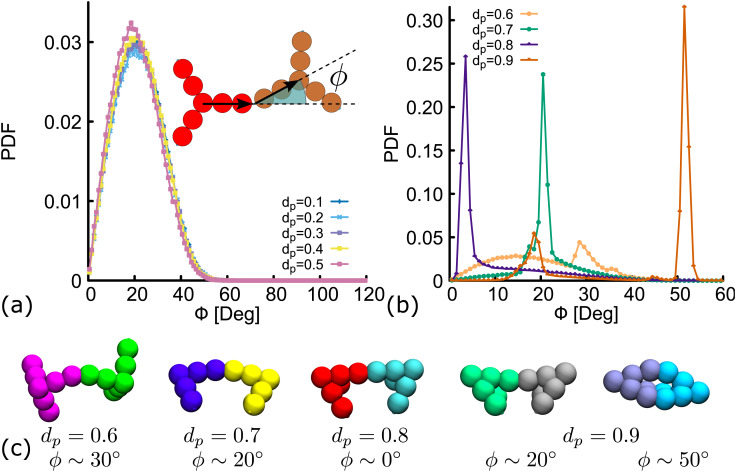
Distribution of the bending angle (*ϕ*) between the arms of two connected nanostars. (a and b) Show results of the distribution for different degree of planarity (indicated in the labels). (c) Hybridized DNAns from simulations, representing the configuration with a bending angle equal to the maxima of the distribution for different values of *d*_p_ = 0.6–0.9.

Importantly, in the snapshots from simulations depicted in Fig. 5(d), the arms through which two DNAns are hybridized, are in general not aligned. There is an effective bending angle (*ϕ*) at the place of connection. In Fig. 6(a) we show that for simulations with *d*_p_ ≤ 0.5, the bending angle displays a broad distribution with average value at around 〈*ϕ*〉 ∼ 20°. It is worth noting here that although this distribution of *ϕ* was not inferred from higher resolution simulations, our model provides some flexibility to tune this parameter: we expect that just as in previous studies with patchy-colloids,^11^ the value of *ϕ* in our coarse-grained model would depend on the patch width. The bigger the patch, the broader the distribution of *ϕ*, but one needs to be careful so as not to break the single-bond-per-patch condition that ensures fixed valence. Interestingly, when *d*_p_ > 0.5 the geometric restrictions in the DNAns design have an important effect in the distribution of the bending angle that is not observed for patchy-colloids: *ϕ* depends on *d*_p_ (see Fig. 6(b)). At *d*_p_ = 0.6 the global maximum of the distribution is located at *ϕ* ∼ 30°. As *d*_p_ increases to 0.7,0.8, the maximum shifts to lower values (*ϕ* ∼ 20°, 0°), indicating that the arms of connected nanostars tend to be more aligned. Finally, when *d*_p_ = 0.9 two maxima appear in the distribution at *ϕ* ∼ 20° and *ϕ* ∼ 50°. Snapshots from simulations of these angles are depicted in Fig. 6(c). The values of *ϕ* are all compatible with the box-like structures reported before in Fig. 4, 5 and the RDF in Fig. S5(c) (ESI†).

## 7. Viscosity

**Fig. 7:**
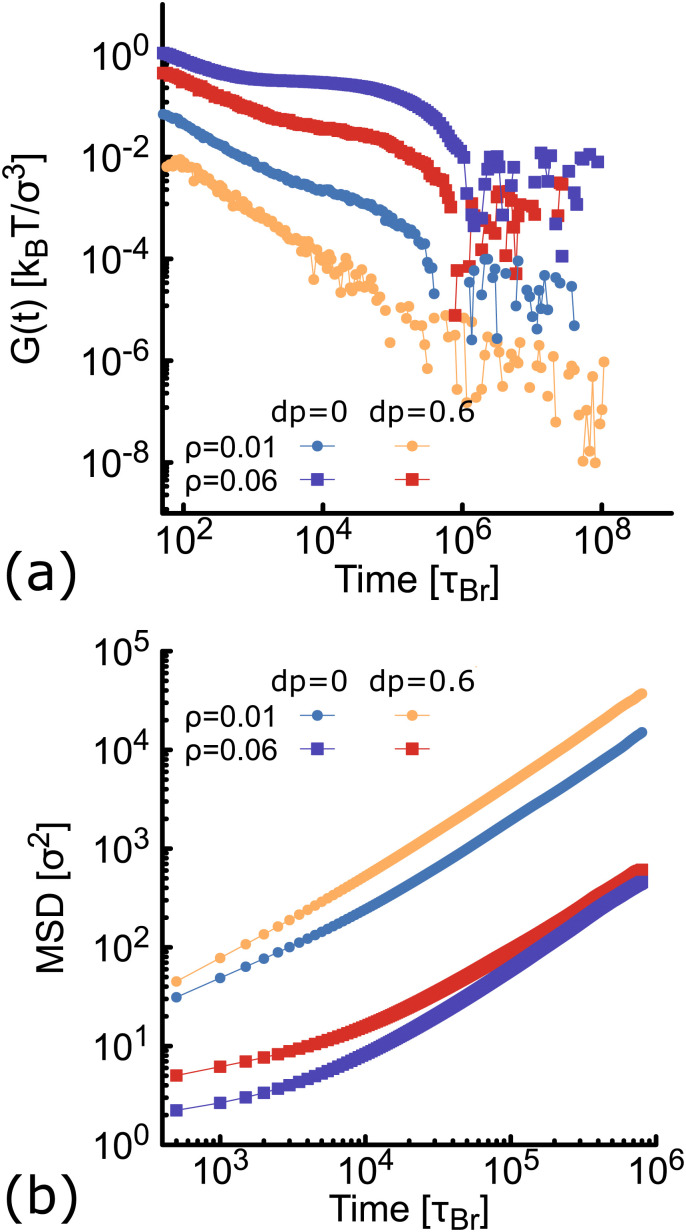
Results from simulations at different concentration of networks formed either by planar (blue and purple) or non-planar (orange and red) molecules (see also Fig. S6 in the ESI†). (a) Autocorrelation (*G*(*t*)) of the stress-tensor from Green–Kubo simulations (see ESI† for details). (b) Log–Log plot of the MSD of DNAns.

Here we compute the zero shear viscosity (*η*) of the planar (*d*_p_ = 0) and non-planar (*d*_p_ = 0.6) networks using the Green–Kubo^36^ relations. This computational method is based in the fact that, transport coefficients like shear and bulk viscosity can be related to the correlation functions of the stress tensor in thermal equilibrium. Simulations were performed at two different volume fractions *ρ* = 0.01 and *ρ* = 0.06, the latter corresponding to a system at the overlapping concentration, made by 1050 DNAns and for which the general results discussed in previous sections hold (see Fig. S6 in the ESI†). In Fig. 7(a) the autocorrelation 
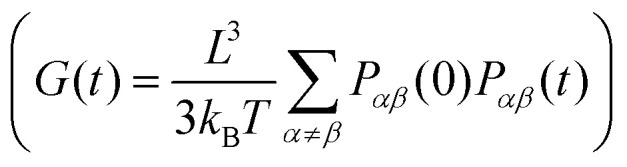
 of the off-diagonal components of the stress-tensor (*P*_*αβ*_ = *P*_*xy*_, *P*_*xz*_ and *P*_*yz*_) is shown. This was computed using the multiple-tau correlator method^37^ from long equilibrium MD simulations (see ESI† for details). The viscosity of the system is then obtained as the integral of *G*(*t*). Keeping in mind that as with any simulation, one needs to be cautious in interpreting units, below we provide a rough estimation of the viscosity in experiments. The networks formed by planar designs have a viscosity *η*_p_ = 573 *k*_B_*Tτ*_Br_/*σ*^3^ = 5.5 Pa s (for *ρ* = 0.01) and *η*_p_ = 265584 *k*_B_*Tτ*_Br_/*σ*^3^ = 2549 Pa s (for *ρ* = 0.06). As comparison, the viscosity of gels at room temperature and low salt concentration, made of tetravalent DNAns and at a concentration of [DNAns] = 220 μM, is *η* ∼ 100 Pa s.^38^ Considering that the latter two conditions affect the viscosity of the system, the simplicity of our model and the different volume fraction used here, our estimate of the viscosity is reasonably. Remarkably, when the networks are formed by non-planar designs, the viscosity is *η*_np_ = 29 *k*_B_*Tτ*_Br_/*σ*^3^ = 0.28 Pa s (for *ρ* = 0.01) and *η*_np_ = 19124 *k*_B_*Tτ*_Br_/*σ*^3^ = 184 Pa s (for *ρ* = 0.06), consistently lower than the one made by planar nanostars. It is worth noting here that the change in viscosity (*η*_p_/*η*_np_ ∼ 20) at a volume fraction of 0.01, is larger than the one at 0.06 (*η*_p_/*η*_np_ ∼ 14). This is in qualitative agreement with the MSD of nanostars reported in Fig. 7(b). At *ρ* = 0.01 the MSD shows a diffusive behaviour, with a considerable gap between non-planar (orange) and planar (blue) networks. As the concentration increases to *ρ* = 0.06, this gap decreases. It is important to stress here that for *ρ* = 0.06 the MSD of planar and non-planar designs displays a subdiffusive regime (at early times), which ends in a freely diffusive regime at late times. This is a typical signature of viscoelastic behaviour that is also captured by the plateau of *G*(*t*) (at *t* ∼ 10^4^*τ*_Br_).

Finally, in the discussion so far, it is clear that the planarity of the DNAns in our model plays a major role in determining the geometry, topology and viscoelasticity of the networks formed. However, one may wonder if this result is related to the fact that we use rigid bodies to represent DNAns. In the ESI† (Fig. S7) we introduce a generalization of our coarse-grained model with more degrees of freedom. In particular, we found that allowing small fluctuations of the angles *α*_*ij*_ between the arms of a DNAns leads to results in good agreement with the discussion in the previous sections.

## 8. Conclusions

In summary, we have introduced a method to characterize the geometry of DNA nanostars from metadynamics simulations and proposed a way to regulate the planarity of DNAns by varying the number of unpaired nucleotides at the core. We used this information to build a coarse-grained model of rigid analogues of nanostars that allows us to explore bulk properties of complex three dimensional percolating networks. Our simulations provide physical insights on how the geometry of DNAns has a major impact on the connectivity of the network and ultimately on the viscoelastic properties of the DNA hydrogels. We found that in gels made of non-planar molecules, box-like structures are formed. This leads to a higher number of contacts, but also to a lower connectivity of DNAns into a single component of the network. As a result, the mobility of molecules is larger in the non-planar case, and a lower viscosity is measured from Green–Kubo simulations. Our results are consistent across different values of *d*_p_ and concentrations. The less-planar the molecules, the larger the number of clusters formed, and therefore, a lower viscosity is computed. At high concentration of DNAns, both *G*(*t*) and the MSD display a viscoelastic behaviour.

We also showed that a different mechanism to control the planarity of DNAns is by changing the salt concentration of the system. However, we anticipate that in these conditions more variables should be considered. The salt concentration would not only modulate the shape of nanostars, but would also make sticky-ends hybridization more stable, increasing the time for network reconfiguration *τ*_c_. We also note that the oxDNA model allows to effectively modulate the electrostatic interaction of the nucleotides by setting the concentration of monovalent salt (sodium chloride). It would be interesting to study the effect of divalent salts, like magnesium chloride, that are commonly used in experiments.

While our coarse-grained model is currently less sophisticated than other mesoscopic models, such as oxDNA, it is also robust enough to capture the overall formation of the network and computationally efficient to probe properties at large volume fractions if desired. Importantly, this model can be also extended to treat DNAns as bead-spring polymers (not longer rigid bodies) and to include fluctuations in the geometry that are expected for real DNAns. It would be interesting to characterize and study the role of this aspect in the future.

## Conflicts of interest

There are no conflicts to declare.

## Supplementary Material

SM-019-D2SM00221C-s001

SM-019-D2SM00221C-s002

SM-019-D2SM00221C-s003
