# Supplementary material for: Nanostars planarity modulates the rheology of DNA hydrogels
Source: Soft Matter. 2023 May 25;19(26):4820–8. doi: 10.1039/d2sm00221c (PMC10321217; doi:10.1039/d2sm00221c)
Supplement: SM-019-D2SM00221C-s001 [file SM-019-D2SM00221C-s001.pdf]

Electronic Supplementary Material (ESI) for Soft Matter.
This journal is © The Royal Society of Chemistry 2023

Soft Matter

ARTICLE TYPE

Cite this: DOI: 00.0000/xxxxxxxxxx

Supplementary Information: Planarity modulates the rheology of DNA hydrogels

Yair Augusto Gutiérrez Fosado,‡[a]

Contents

I Details of Metadynamics simulations 2
Reminder of the theory . . . 2
The collective variable . . . 2
The oxDNA model . . . 3
Implementation . . . 3

II Coarse-Grained Molecular Dynamics Simulations 3

III Network formation at fixed concentration 4

IV Structural analysis 5

V Varying d_p 5

VI Green-Kubo relations 6

VII Varying concentration 6

VIII Non-rigid model 7

[a] *School of Physics and Astronomy, University of Edinburgh, Peter Guthrie Tait Road, Edinburgh, EH9 3FD, UK.*

I Details of Metadynamics simulations

Reminder of the theory

As described in the main text, Metadynamics (MetaD) is a computer simulation method that can be used to estimate the free energy landscape (FEL) of a system. Importantly, it is assumed that this FEL can be written in terms of few Collective variables (CVs), $\zeta_i(\mathbf{r})$, that depend only on the positions, $(\mathbf{r})$, of particles and that can distinguish between any two (or more) states of the system. Different states are represented by minima in the FEL.

In MetaD, the sampling of the phase-space of a system is facilitated by the introduction of a history-dependent bias potential that forces the system to migrate from one minimum of the FEL to the next one. When just one CV is considered the potential, constructed as a sum of Gaussians at time t, has the form:

$$U_G(\zeta(\mathbf{r}),t) = \sum_{t'=\tau_G,2\tau_G,\ldots} B\exp\left(-\frac{[\zeta(\mathbf{r})-\nu(t')]^2}{2\Delta^2}\right), \quad \text{(S1)}$$

where B,Δ and τ_g are constants representing the Gaussian height, width and frequency at which the Gaussians are added, respectively. $\nu(t') = \zeta(\mathbf{r}(t'))$ represents the the value of the CV at time t', and $t' < t$ must be satisfied. If B is large, the free energy surface will be explored at a fast pace, but the reconstructed profile will be affected by large errors. Instead, if B or τ_g are small the reconstruction will be accurate, but it will take a longer time.

The basic assumption of metadynamics is that after a sufficiently long time, $\lim_{t\to\infty} U_G(\zeta,t)$ provides an estimate of the free energy landscape, $F(\zeta)$, modulo a constant. However, since the potential of the system changes every time a Gaussian is added, $U_G(\zeta,t)$ oscillates around $F(\zeta)$ at long times. Another method that ensures that the FEL converges more smoothly is the Well-Tempered Metadynamics (WTMetaD). In this variant of MetaD, the height of the Gaussians added is no longer a constant but it scales at each timestep, so the bias potential is obtained by replacing B in Eq. S1 by:

$$\omega = B\exp\left[-U_G(\zeta(\mathbf{r}(t')),t')/\Delta T\right], \quad \text{(S2)}$$

where ΔT has the dimension of temperature. This parameter is related to the bias factor ($\gamma = (T+\Delta T)/T$), which indicates how fast the Gaussian height decreases in a simulation. The higher the value of γ, the slower the height decrease. Therefore, $\gamma \to \infty$ represents normal MetaD simulations and $\gamma = 1$ represent ordinary molecular dynamics (MD) simulations.

The collective variable

Here we define a collective variable (d_p, described in the main text) that can distinguish between planar and non-planar geometries of the DNAns. In practice, d_p is obtained from the direction of each of the dsDNA arms in a nanostar. This is done in the following way: First we compute the position, $\mathbf{r}_{core}$, of the DNAns core. This is done by locating the base-pair (bp) closer to the FJC in each arm (three in total) and calculating the centre of mass (COM) using the position of the six nucleotides previously selected. Then, the unitary vectors $(\hat{e}_1,\hat{e}_2,\hat{e}_3)$ pointing from the core of the molecule to the COM of the bp closer to the sticky end in each arm, define the direction of the three dsDNA arms.

To compute d_p we find two vectors ($\mathbf{A}$ and $\mathbf{B}$) that connect the end of any two arms, for example:

$$\mathbf{A} = \hat{e}_2 - \hat{e}_1.$$

$$\mathbf{B} = \hat{e}_3 - \hat{e}_1. \quad \text{(S3)}$$

These vectors define a plane whose normal can be found as the cross product $\mathbf{n} = \mathbf{A}\times\mathbf{B}$. Then, the distance d_p from this plane

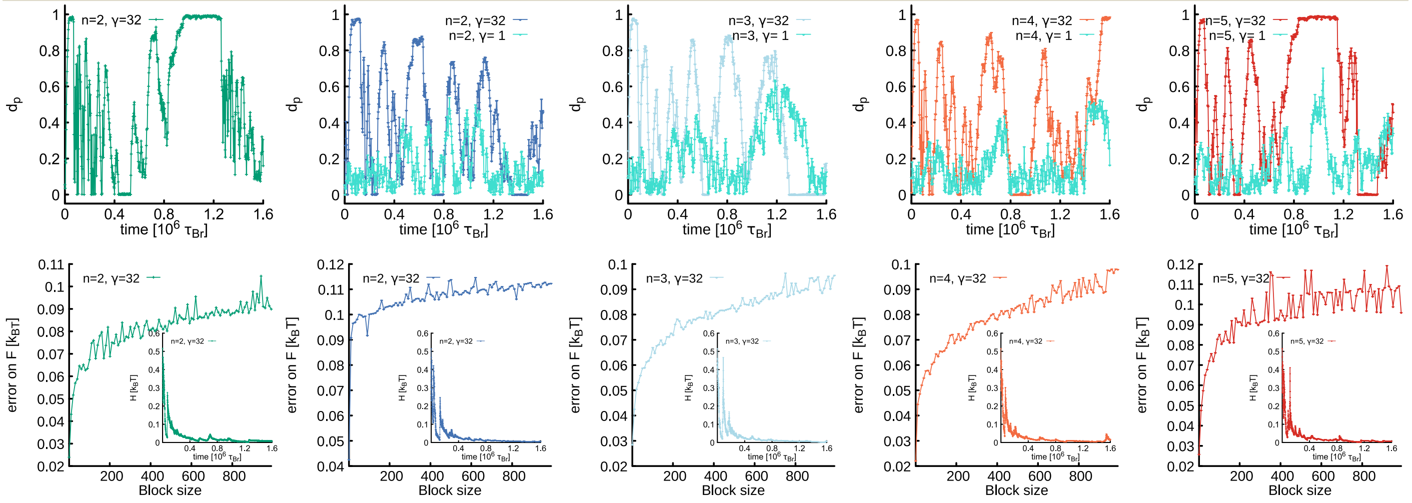

Fig. S1 Metadynamics simulations. First column shows result at a high salt concentration [NaCl]=1 M for $n = 2$ unpaired nucleotides at the FJC. From column two to five we show results at [NaCl]=0.15 M for $n = 2,3,4$ and 5, respectively. Top plots show the time evolution of the collective variable (d_p). In each plot, two curves with different colour are shown. In turquoise we show results for a normal MD simulation without bias ($\gamma = 1$) and the other colour represents results from WTMetaD simulations with $\gamma = 32$. Bottom depicts the error analysis on d_p as function of the block size. Insets show the temporal evolution of the Gaussian Height for the WTMetaD

to the core of the molecule, is given by the projection of any of the three dsDNA arms (for example $\hat{\boldsymbol{e}}_2$) onto the unitary normal vector:

$$d_p = |\hat{\boldsymbol{e}}_2 \cdot \hat{\boldsymbol{n}}| . \tag{S4}$$

The range of our CV is from $d_p = 0$ (for a completely planar DNAns) to $d_p \lesssim 1$. The larger the value of d_p the less planar the molecule. Note that d_p cannot be equal to 1 due to the excluded volume interaction between arms.

The oxDNA model

We used the most recent implementation[1] of the oxDNA model into LAMMPS[2] (Large Scale Molecular Massively Parallel Simulator). Briefly, this model describes DNA at the single nucleotide level by means of a rigid body with additive-pairwise interaction sites. The potentials involved in the interactions accurately represent: the hydrogen bonding between complementary bases, the connectivity of the sugar-phosphate backbone, the excluded volume between nucleotides and also the stacking, coaxial-stacking and cross-stacking interactions. The relation between one simulation unit (SU) in the oxDNA and the international system (SI) units is: mass ($M = 100AMU = 1.66 \times 10^{-25} kg$), temperature ($T = 3000K$), length ($L_s = 8.518 \times 10^{-10} m$), energy ($\epsilon_s = k_B T = 4.142 \times 10^{-20} J$) and force ($F = \epsilon_s / L_s = 4.863 \times 10^{-11} N$). The simulation time $\tau_{LJ} = L_s \sqrt{M/\epsilon_s} = 1.7 ps$, comes naturally from the above quantities and it is employed to define a constant integration timestep $\delta t = 0.001 \tau_{LJ}$.

Implementation

Here we use the plumed[3] library implemented in LAMMPS to perform Well Tempered Metadynamics of a three-armed DNA nanostar simulated with the oxDNA model. The sequence of this molecule is given in Table 1 of the main text and it corresponds to the one with $n = 2$ unpaired nucleotides at the FJC. We also performed WTMetaD to molecules with $n = 3, 4$ and 5 (see Fig. S1). The parameters used in the WTMetaD simulations are: $\gamma = 32$, $B = 0.5 k_B T$, $\Delta = 0.05\sigma$ and $\tau_g = 4 \times 10^5$ timesteps. Each simulation was run for a total of 1.6×10^9 timesteps.

As described above, by choosing carefully the values of the bias factor (γ) in the WTMetaD we can compare results from different methods, namely, MD ($\gamma = 1$) and WTMetad ($\gamma = 32$). Top plots in Fig. S1 show the time evolution of d_p. We observe that in simulations without the bias potential (shown in turquoise) d_p does not explore the full range of possible values (d_p is defined in the interval [0,1]). The maximum value reached is ~ 0.65, it only happens briefly and for $n = 5$. Instead, in MetaD the CV is able to explore the whole range of configurations. Bottom plots show the error block analysis of the FEL, which is used to assess the convergence of the WTMetaD simulations. As expected, the error increases with the block size until it reaches a plateau in correspondence of a dimension of the block that exceeds the correlation between data points.

II Coarse-Grained Molecular Dynamics Simulations

Molecular dynamics simulations are performed using the model described in the main text. Here we refer to the details on those simulations. The Langevin integration of the system was carried out using LAMMPS in an NVT ensemble by a standard velocity-Verlet algorithm with integration time-step $\delta t = 0.01$. The position of particles in the system obeys the following equation

$$m \frac{d^2 \mathbf{r}}{dt^2} = -\xi \frac{d\mathbf{r}}{dt} - \nabla U + \sqrt{2 k_B T \xi} \Lambda(t), \tag{S5}$$

where $m = 1$ is the mass of the particle, $\mathbf{r}$ represents its position. ξ is the friction and $\Lambda(t)$ is the white noise term with zero mean which satisfies $\langle \Lambda_\alpha(t) \Lambda_\beta(s) \rangle = \delta_{\alpha\beta} \delta(s-t)$ along each Cartesian coordinate represented by the Greek letters. The total potential field experience by a particle is U and depends on the interactions set for a particular system.

In our coarse-grained model, each DNA nanostars is represented by a rigid body made up by ten particles. Seven beads account for the DNAns core and their excluded volume is modelled via a truncated and shifted Lennard-Jones (LJ) potential:

$$U_{LJ}(r) = 4\epsilon \left[\left(\frac{\sigma}{r}\right)^{12} - \left(\frac{\sigma}{r}\right)^{6} + \frac{1}{4} \right], \tag{S6}$$

if $r < 2^{1/6}\sigma$, and $U_{LJ}(r) = 0$ otherwise. Here $\sigma = 2.5$ nm represents the diameter of a spherical bead, $\epsilon = 1.0$ parametrises the strength of the repulsion and r is the Euclidean distance between the beads.

Patches are placed at a distance of 2.5σ from the core of the molecule and on the surface of the outermost bead along each arm. The sticky ends interaction, responsible for holding two DNAns together, is modelled by a Morse potential:

$$U_m(r) = \epsilon_m \left[e^{-2\alpha_0 (r - r_0)} - 2 e^{-\alpha_0 (r - r_0)} \right], \tag{S7}$$

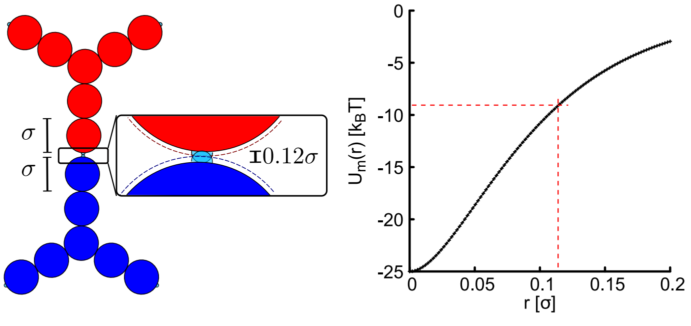

Fig. S2 Left panel shows schematic representation of two bound DNAns. The hard-sphere repulsion between beads forces patches to remain at a minimum distance of 0.12 σ. Right panel shows the plot of the morse potential used in simulations to set the attraction between patches (obtained from Eq. S7 with $\epsilon_m = 25.0 k_B T$, $\alpha_0 = 14\sigma^{-1}$ and $r_0 = 0$). Although the equilibrium distance between patches is set to zero, the minimum distance allowed by the excluded volume of the beads sets the energy $U_m(r = 0.12\sigma) \sim 10\ k_B T$ (red dashed lines).

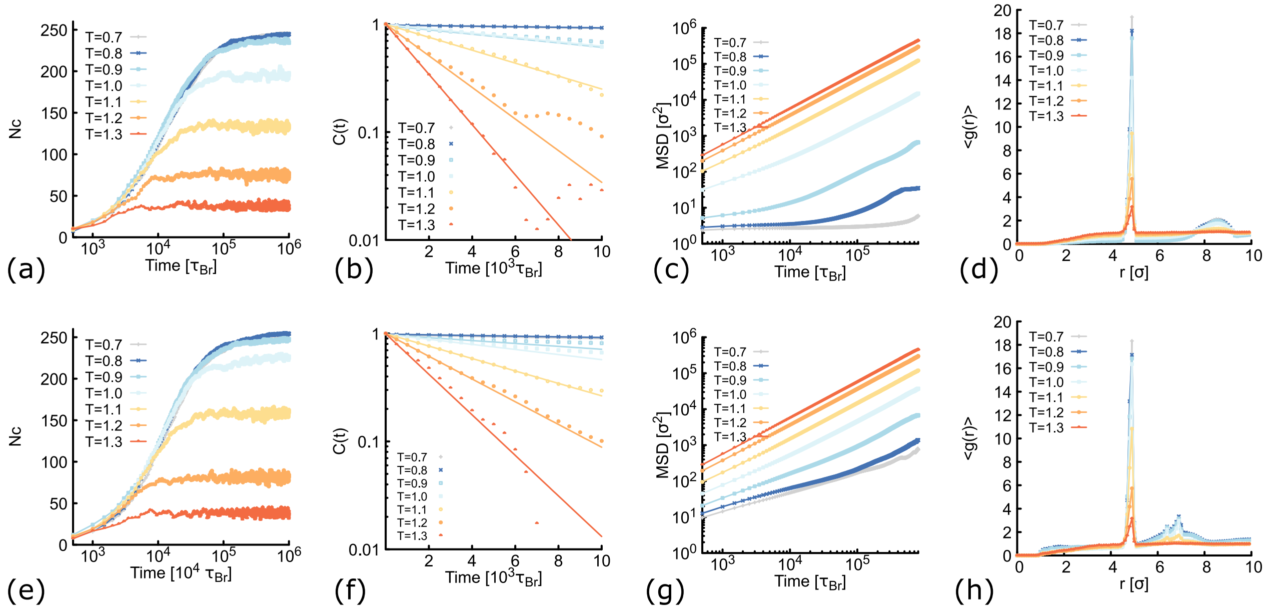

Fig. S3 Time evolution of different quantities computed from simulations of networks with planar ($d_p = 0$, top panels) and non-planar ($d_p = 0.6$, bottom panels) molecules at a fixed volume fraction $\rho = 0.01$. Different colours represent different temperatures (quoted in units of ϵ/k_B). (**a,e**) show examples of trajectories obtained during the network formation. The curves represent the number of contacts N_c at a given time t. In order to compute the fraction of connected patches $\vartheta(t)$, we have to multiply N_c by the normalization factor $2/Nf$. (**b,f**) Log-linear plot of the autocorrelation function of $N_c(t)$ computed from Eq.S9. Dots represent results from simulations and lines are a fit to an exponential decay, with decay constant τ_c. (**c,g**) show log-log plots of the MSD averaged over all the molecules in the system. (**d,h**) Radial distribution function averaged over configurations at long times (when the network is formed).

for $r < R_c$. Here, r represents the distance between patches of two adjacent nanostars, $r_0 = 0$ is their equilibrium distance and $R_c = 0.2\sigma$ is the cut-off distance of attraction. The amplitude of the potential is set to $\epsilon_m = 25.0 k_B T$ and $\alpha_0 = 14\sigma^{-1}$ controls the width of the potential. These parameters were chosen to ensure that during the simulation any of the three arms of one ns can hybridize with any (but only one) of the arms of another ns. Importantly, the centre of a patch is placed at the edge of the last bead forming a dsDNA arm, i.e., at a distance of 0.5 σ from the centre of that bead. Since the hard-sphere repulsion of beads (set by the LJ potential in Eq. S6) covers a radius of $(2^{1/6}\sigma)/2 = 0.56\sigma$, we expect hybridized patches to be at a minimum distance of $\sim 0.12\sigma$. Therefore, the binding energy felt by hybridized patches is $\sim 10 k_B T$ (see Fig.S2), in agreement with the activation energy E_a mentioned in the main text.

It is worth mentioning here that in the CG model we quote time in units of τ_{Br}, the Brownian time, which is proportional to the time needed for a DNA bead to diffuse its own size. Finally, we note that the total excluded volume (V_1) of one DNAns is given by the sum of the volume of all its structural beads:

$$V_1 = 7\frac{\pi\sigma^3}{6} \tag{S8}$$

In all our simulations the initial configuration is obtained by placing N nanostars in a cubic simulation box of length $L = 40\sigma$ and with periodic boundary conditions. The volume fraction of this system is $\rho = NV_1/L^3$. We set $\epsilon_m = 0$ so nanostars cannot hybridized and we equilibrate the system for 5×10^5 τ_{Br}. We then turn on the attraction between patches and record the evolution of our system for 10^6 τ_{Br}. Results for planar and non-planar nanostars at different temperatures are shown in Fig. S3.

III Network formation at fixed concentration

Melting curve

In the main text we show the melting profiles $\langle\vartheta\rangle$ as a function of the temperature for the system at $\rho = 0.01$ (Figure **3**(a)). This is obtained by averaging over the last 2 10^5 τ_{Br} of the trajectories, the number of contacts $N_c(t)$ (multiplied by the normalization factor $2/Nf$). Examples are shown in Figs. S3(a,e). It is clear that all the samples start from an equilibrated configuration of unconnected nanostars ($N_c(0) = 0$); after turning on the attraction between patches the system evolves to a new steady state where a network is formed. The higher the temperature the more unconnected the stars and the smaller the value of $< \vartheta >$.

The autocorrelation function

From the trajectory of N_c at long times, it is also possible to compute the time for network reconfiguration (τ_c). This is done by first finding the autocorrelation function of $N_c(t)$, which in the discrete case has the form:

$$c(t) = \frac{\sum_{t=0}^{t_f - t'} (N_c(t) - \mu)(N_c(t+t') - \mu)}{\sum_{t=0}^{t_f} (N_c(t) - \mu)^2}, \tag{S9}$$

where t_f is the total number of time data points, t' is the lag time and μ is the mean of the data. Results at different temperatures are shown in Figs. S3(b,f). The autocorrelation function follows

an exponential decay $c(t) \propto e^{-t/\tau_c}$, from which τ_c is computed. In the main text we show τ_c as function of T in Figure **3**(b). We also note that, as expected, the data at lower temperatures has larger correlations. This is in agreement with the MSD plots shown in Figs. S3(c,g), where the system at lower temperatures has a smaller mobility.

IV Structural analysis

The radial distribution function

The structure of our networks is analysed by computing the radial distribution function (RDF) of beads at the core of the DNA nanostars:

$$g(r) = \frac{1}{4\pi \rho_{av} M} \sum_{i=1}^{M} \sum_{j\neq i}^{M} \langle \delta(|r_{ij} - r|) \rangle, \tag{S10}$$

where $\rho_{av} = N/L^3$ is the averaged density of core beads in the simulation, r_{ij} represents the distance between the cores of molecules i and j. The sum counts the number of DNAns cores that are at a distance r.

In our simulations at fixed $\rho = 0.01$ there is a total of $N = 175$ nanostars (and core beads). We compute $g(r)$ using the wrapped coordinates in the following way: (i) First, we choose the M core beads that are inside an sphere of radius $R_{select} = L/4$ with centre at the origin of the box. (ii) We loop over each one of this M particles, and we count the number of core beads neighbours that are at a distance r. This search radius can cover up to a distance $L/4$ and in the count we consider all the core beads in the simulation (not only the M selected in (i)). (iii) We use this information in Eq. S10 to compute $g(r)$. It is worth noting here that we repeat this procedure to compute $g(r)$ in all the configurations in the steady state (at long times and when $\vartheta(t)$ has reached a plateau) and we take the average over these configurations.

Figs. S3(d,h) show the average RDF obtained at different temperatures for planar and non-planar molecules, respectively. In both cases, $g(r)$ shows a maximum located near to 5σ, corresponding to the average distance between the cores of two bound nanostars. As temperature increases, the hight of this maximum decreases, meaning that there are less contacts between nanostars.

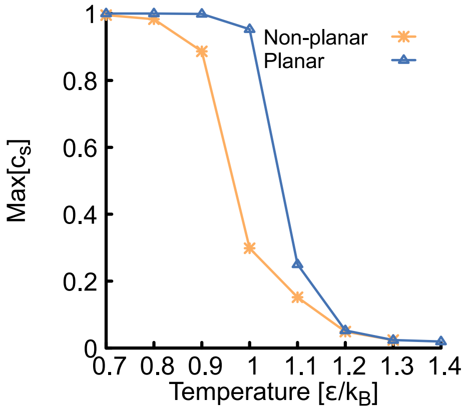

Fig. S4 Number of connected nanostars in the largest cluster of the network (normalized by the total number of DNAns) as function of the temperature for planar ($d_p = 0$, in blue) and non-planar ($d_p = 0.6$, in orange) molecules. All the data shown here correspond to simulations performed at $\rho = 0.01$

Largest cluster in the network

Figures S4 shows the size of the largest cluster in the networks (normalized by the number of nanostars N=175) made of either, planar or non-planar molecules. Over the whole range of temperatures explored, the size of the largest cluster (Max(C_s)) is larger in the case of networks made out of planar molecules.

V Varying d_p

In the main text we show the fraction of connected DNAns $\langle \vartheta \rangle$ and the relaxation time τ_c as function of the planarity (d_p), when simulations are performed at a fixed temperature ($T = 1.0\epsilon/k_B$)

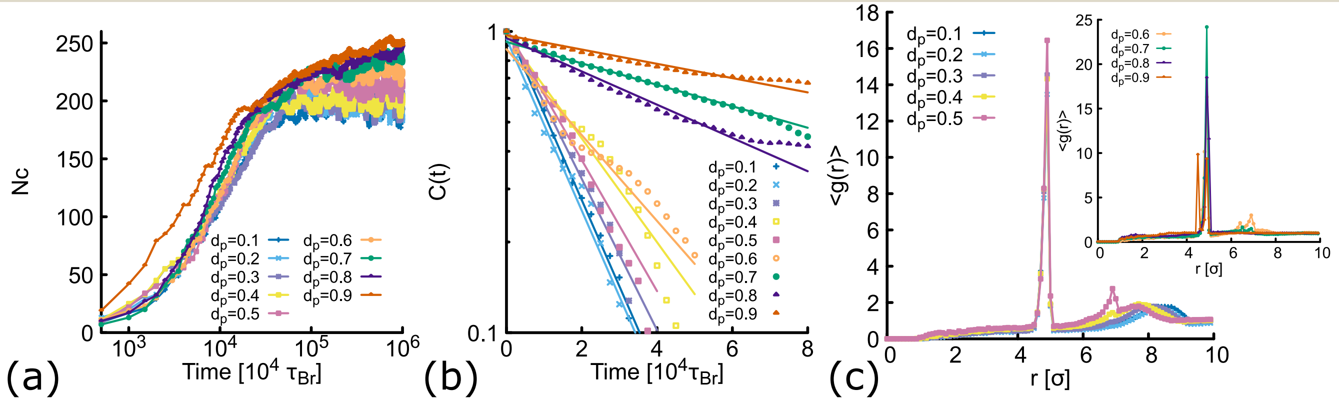

Fig. S5 Time evolution of different quantities computed from simulations of DNAns with different degree of planarity at $\rho = 0.01$ and fixed temperature $T = 1.0\epsilon/k_B$. Different colours represent different planarity: from $d_p = 0.1$ to $d_p = 0.9$. In orange we show results for $d_p = 0.6$, the one used so far as non-planar in the main text and ESI. (**a**) shows trajectories obtained during the network formation. The curves represent the number of contacts N_c at a given time t. (**b**) Log-linear plot of the autocorrelation function of $N_c(t)$ computed from Eq.S9. Dots represent results from simulations and lines are a fit to an exponential decay, with decay constant τ_c. (**c**) Radial distribution function averaged over configurations at long times (when the network is formed). Main plot shows results for $dp \leq 0.5$ and inset shows results for $d_p > 0.5$.

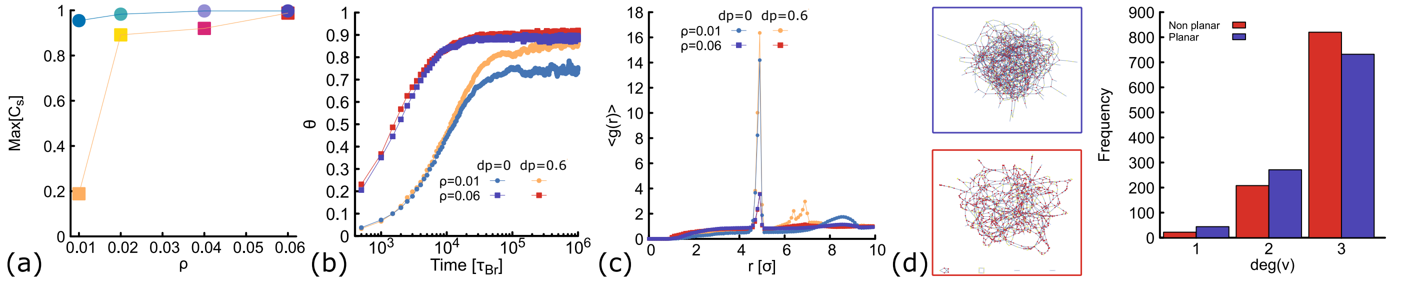

Fig. S6 Results from simulations of planar ($d_p = 0$) and non-planar ($d_p = 0.6$) nanostars at a fixed temperature ($T = 1.0\epsilon/k_B$) and at different concentrations. (**a**) Largest connected component as function of volume fraction. Circles and squares represent results at $d_p = 0$ and $d_p = 06$, respectively. Lines are a guide to the eye. (**b**)-(**d**) compare results at $\rho = 0.01$ and $\rho = 0.06$. (**b**) shows trajectories obtained during the network formation. The curves represent the fraction of contacts at a given time t. (**c**) Radial distribution function averaged over configurations at long times (when the network is formed). (**d**) Network diagrams (left) and histogram (right), showing the connectivity between planar (red) and non-planar (purple) DNAns at $\rho = 0.06$.

and concentration ($\rho = 0.01$). Here we provide the full trajectories from which these results were obtained. Fig. S5(a-b) show the temporal evolution of $N_c(t)$ and $c(t)$. We observe that as d_p increases, more contacts between DNAns are formed and the contacts-correlation is larger. The RDF in Fig. S5(c) shows that the two local maxima located at $r \in [6,8]$ are developed when d_p reaches values $\in [0.5, 0.6]$. At higher values of d_p, the amplitude of $g(r)$ at these local maxima shrinks, and the amplitude of the global maximum (at r close to 5) increases. This is consistent with the increase in the number of box-like structures observed in the network diagrams of Fig. **5** in the main text. In the extreme case $d_p = 0.9$, one local maxima appears at particular small values of $r < 5$. This is in agreement with the structure made by four DNAns and depicted in the snapshot of Fig. **5**(d) in the main text and for which DNAns connect with a bending angle $\phi \sim 20^{\circ}$, as shown in Fig. **6**(c).

VI Green-Kubo relations

The autocorrelation function ($G(t)$) is defined as:

$$G(t) = \frac{L^3}{3k_BT} \sum_{\alpha \neq \beta} P_{\alpha\beta}(0) P_{\alpha\beta}(t) \tag{S11}$$

where $P_{\alpha\beta}$ represents the out-off diagonal component (P_{xy}, P_{xz} and P_{yz}) of the stress tensor. Results from long simulations ($1.6 \times 10^6 \tau_{Br}$) are shown in Fig. **4**(c) of the main text. The autocorrelation was computed using the multiple-tau correlator method described in reference [4] and implemented in LAMMPS with the *fix ave/correlate/long* command. This computation ensures that the systematic error of the multiple-tau correlator is always below the level of the statistical error of a typical simulation (see LAMMPS documentation).

The viscosity (η) of the system is then obtained by computing the following integral

$$\eta = \int_0^{t \to \infty} G(t) \mathrm{d}t. \tag{S12}$$

in practice we compute the numerical discrete integral of the curves in Fig. **4**(c). We obtained the viscosity in simulation units for planar ($\eta_p = 573\ k_BT\tau_{Br}/\sigma^3$) and non-planar ($\eta_{np} = 29\ k_BT\tau_{Br}/\sigma^3$) networks at $\rho = 0.01$. To convert these results to real units we use $\sigma = 2.5$ nm, $k_BT = 4.11\ pN \cdot nm$ and the Brownian time:

$$\begin{aligned} \tau_{Br} &= \sigma^2/D \\ &= \sigma^2/\mu_s k_B T \\ &= 3\pi\eta_s\sigma^3/k_BT. \end{aligned} \tag{S13}$$

Assuming that the solvent is water (with viscosity $\eta_s = 1\ mPa \cdot s$) we obtain $\tau_{Br} = 36$ ns. Therefore, the viscosities of the two networks map to $\eta_p = 5.5 Pa \cdot s$ and $\eta_{np} = 0.28 Pa \cdot s$.

VII Varying concentration

Up to now, we have presented results from simulations performed at a constant volume fraction $\rho = 0.01$. In this section, we investigate the effect of increasing concentration for networks made of planar and non-planar nanostars. Without loss of generality we choose $d_p = 0.6$ for the non-planar case. First, we compute the size of the largest connected component of the network (Max(C_s)). When Max(C_s)= N, there is only one cluster in the system that is formed by the connection of all DNAns. In contrast, when the value of Max(C_s)< N, nanostars are split into clusters. In Fig. S6(a) we report the value of Max(C_s) obtained from simulations at different volume fractions, note that the results are normalized by the number of nanostars at each concentration. We observe that as ρ increases, the fraction of nanostars connected to the largest cluster also grows, for both planar and non-planar molecules. However, the value of Max(C_s) is consistently lower in the non-planar case. At $\rho = 0.06$, most of the nanostars are part of a single cluster regardless their planarity. In the following, we focus in results obtained at this value of ρ, but the same general results were obtained at different volume fractions.

In Fig. S6(b) we corroborate that at a fixed volume fraction (either $\rho = 0.01$ or $\rho = 0.06$), the equilibrium value of the fraction of contacts (θ) is larger for non-planar nanostars. The RDF is shown in Fig. S6(c), we observe that although the height of the peaks has decreased with the concentration, the region at which the maxima appear is conserved (compare blue and purple, or

orange and red). Finally, Fig. S6(d) shows the network diagrams obtained for both designs. For the non-planar case a few small clusters appear, indicating a slightly lower connectivity than the planar case. The histogram depicting the frequency of the nanostars with certain degree of connection is also shown. As it can be seen, the number of DNAns fully connected ($deg(v) = 3$) is larger for the non-planar molecules. This is all in agreement with the discussion in the main text, where we show results mainly for $\rho = 0.01$.

VIII Non-rigid model

In the discussion so far, it is clear that the planarity of the DNAns plays a major role in determining the geometry and topology of DNA hydrogels, and in consequence, the planarity also determines their viscoelastic properties. However, one may wonder if this result could be somehow related to the fact that we use rigid bodies to represent DNAns in our coarse-grained model. Here we first introduce a coarse-grained model of DNAns with additional degrees of freedom, and then we use it to prove that our results hold when we allow small fluctuations of the angle α_{ij} between the dsDNA arms of individual nanostars.

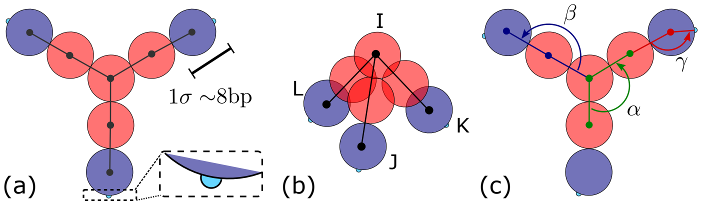

Fig. S7 Sketch of the interactions in the coarse-grained non-rigid model. (**a**) shows the particles used: beads (red) and patchy-beads (blue with cyan patches). Lines represent the harmonic bonds connecting two beads. (**b**) The planarity of the model is controlled with an improper dihedral. (**c**) The three type of angle interactions used to give structure to the DNA nanostars.

The non-rigid model of DNAns is depicted in Fig. 7 and it can be seen as a generalization of the model used in the main text. It consists of four beads (depicted in red) forming the core of the molecule, and three beads (depicted in blue) with a patch attached to their edge (depicted in cyan). Each of these patchy-beads is integrated as a rigid body. All beads have an excluded volume of 1σ modelled with the LJ potential in Eq. S6. The attraction between patches is modelled with the Morse potential in Eq. S7. Neighbouring beads are connected via a harmonic potential:

$$U_{bond} = \epsilon_{bond}(r - r_0)^2, \tag{S14}$$

where $r_0 = 1.1224\sigma$ is the equilibrium distance between beads and $\epsilon_{bond} = 300k_BT$ holds beads in place and only allows for small fluctuations around r_0.

We use an improper dihedral (called *umbrella* style in LAMMPS) to have control over the planarity of the DNAns. The potential representing this interaction has the form:

$$U_{imp} = \frac{\epsilon_{imp}}{2}\left(\frac{1}{\sin(w_0)}\right)^2[\cos(w) - \cos(w_0)]^2, \text{ for } w_0 \neq 0^\circ,$$
$$= \epsilon_{imp}[1 - \cos(w)] \text{ for } w_0 = 0^\circ, \tag{S15}$$

where w is the angle between the IL axis and the IJK plane in Fig. 7(b). $\epsilon_{imp} = 300k_BT$ is the stiffness of the potential imposing an equilibrium angle w_0. For planar molecules we set $w_0 = 0^\circ$ and for non-planar molecules we set $w_0 = 87^\circ$, which corresponds to $d_p \sim 0.6$.

The angle interactions are sketched in Fig. 7(c): α represents the angle between any two arms of a nanostar, β is the angle used to align the beads forming a single dsDNA arm, and γ is the angle setting the alignment of the patch with the dsDNA arm. Note that a total of nine angles are needed (3 of each type) to represent a single DNAns. All the angle interactions are modelled by harmonic potentials with equilibrium values $\alpha_0 = 120^\circ$, $\beta_0 = 180^\circ$ and $\gamma_0 = 180^\circ$ to resemble the structure of a DNAns. Because each of the dsDNA arms is $20 - 30$ base-pairs long, smaller than $l_p = 150$ bp (the persistence length of DNA), we expect arms to be straight, and therefore, we set $\epsilon_\beta = 300k_BT$. We decided to set $\epsilon_\gamma = 300k_BT$ to allow for small fluctuations of the alignment of the patches with the arm. Recall that this choice provides a natural distribution of bending angles (ϕ) at the place of hybridization between two DNAns (see Fig. **6** of the main text). To study the impact of the fluctuation of the angles between arms within a single DNAns, we use two different values of ϵ_α. At high values ($\epsilon_\alpha = 300k_BT$), we expect a similar behaviour to the rigid analogues of nanostars studied in the main text. On the other hand, for small values of ϵ_α, nanostars fluctuate around the Y-shaped conformation in a qualitatively compatible way with the conformations found in simulations with the oxDNA model for the planar design (with $n = 2$ at [NaCl]=0.15 M, see for instance Supplementary Movie 2). In practice, we performed simulations with the non-rigid model using $w_0 = 0^\circ$ (planar), and we decreased the value of ϵ_α until we observed that for $\epsilon_\alpha = 6k_BT$, nanostars are able to deform into other conformations.

In Fig. S8 we show results comparing the different models: rigid (used in the main text), non-rigid with $\epsilon_\alpha = 300k_BT$ and non-rigid with $\epsilon_\alpha = 6k_BT$. As we expect, the two first models produce outcomes that are very similar. This is particularly truth for the planar DNAns, where the red and orange curves are very close in Panels (a)-(d). For the non-planar case, curves in blue and cyan deviate a little bit. We attribute this to the fact that $w_0 = 87^\circ$ doesn't provide exactly the value of $d_p = 0.6$. We also note that for the non-rigid model the RDF shows a shift of the position of the local maxima.

When we compare the results for the non-rigid model with the same planarity, the case with $\epsilon_\alpha = 6k_BT$ is more dynamic. Unsurprisingly, the MSD shows that the mobility is lower when $\epsilon_\alpha = 300k_BT$ compared to the result for $\epsilon_\alpha = 6k_BT$. This is also reflected in the autocorrelation of the stress tensor, where $G(t)$ is lower for the latter case.

Finally, Panel (e) displays results for the non-rigid model with $\epsilon_\alpha = 6k_BT$ and it shows that for non-planar molecules, the mobil-

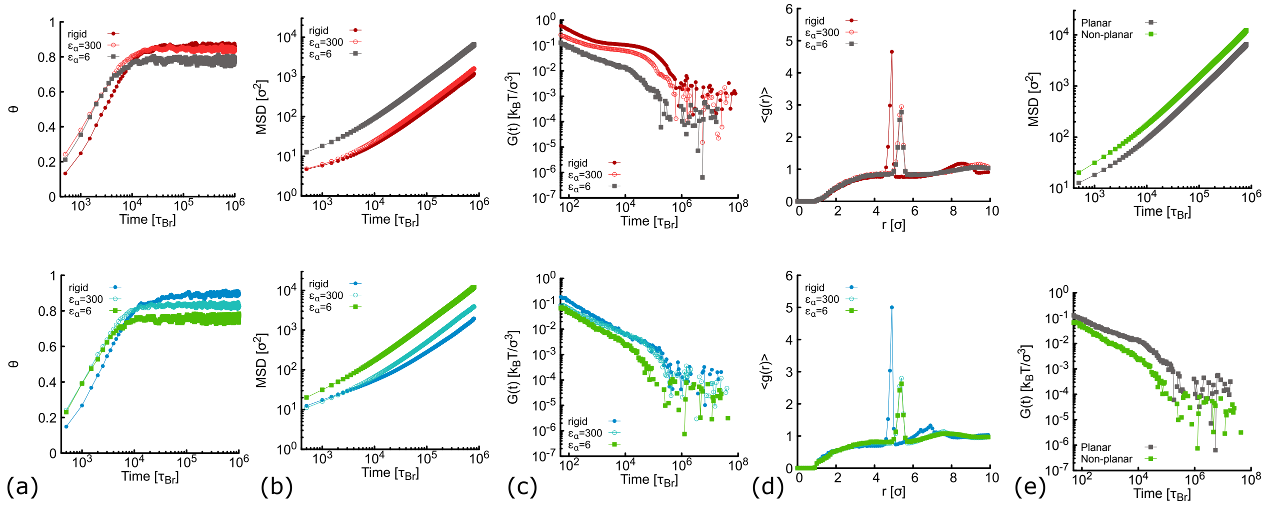

Fig. S8 Comparison of results from simulations performed at $\rho = 0.04$, $T = 1.0k_BT$ for planar (top panels) and non-planar (bottom panels). Colours depict results for the rigid model (red and blue), and the non-rigid model with $\epsilon_\alpha = 300k_BT$ (orange and cyan) and $\epsilon_\alpha = 6k_BT$ (gray and green). (**a**)-(**d**) show the fraction of contacts, MSD, G(t) and the RDF, respectively. (**e**) Comparison of results for planar and non-planar molecules, using the non-rigid model with $\epsilon_\alpha = 6k_BT$.

ity is higher (top) and the viscosity is lower (bottom), than those of the networks formed by planar molecules. This supports the results obtained with the rigid model and allows us to be confident that allowing fluctuations of the angle between arms in a single DNAns, will not change the general results found in the main text. It would also be interesting to perform additional simulations (with higher resolution models of DNA) using the methods described in this manuscript, in order to tune the rest of the potentials in our non-rigid model.

Notes and references

1 O. Henrich, Y. A. G. Fosado, T. Curk and T. E. Ouldridge, *Eur. Phys. J. E*, 2018, **41**, 57.

2 S. Plimpton, *J. Comp. Phys.*, 1995, **117**, 1–19.

3 G. A. Tribello, M. Bonomi, D. Branduardi, C. Camilloni and G. Bussi, *Computer Physics Communications*, 2014, **185**, 604 – 613.

4 J. Ramírez, S. K. Sukumaran, B. Vorselaars and A. E. Likhtman, *The Journal of Chemical Physics*, 2010, **133**, 154103.
